# Phylogenetic relationships and status of taxa of *Pulsatilla uralensis* and *P. patens* s.str. (Ranunculaceae) in north-eastern European Russia

**DOI:** 10.3897/phytokeys.162.53361

**Published:** 2020-10-09

**Authors:** Olga E. Valuyskikh, Ludmila V. Teteryuk, Yana I. Pylina, Oleg E. Sushentsov, Nikita A. Martynenko, Dmitry M. Shadrin

**Affiliations:** 1 Institute of Biology of Komi Scientific Center of Ural Branch of Russian Academy of Sciences, Kommunisticheskaya 28, Syktyvkar, 167982, Russia Komi Scientific Center, Ural Branch of Russian Academy of Sciences Syktyvkar Russia; 2 Botanical Garden of Ural Branch of Russian Academy of Sciences, 8 Marta 202a, Yekaterinburg, 620144, Russia Botanical Garden, Ural Branch of Russian Academy of Sciences Yekaterinburg Russia; 3 K.A. Timiryazev Institute of Plant Physiology of Russian Academy of Sciences, Botanicheskaya 35, Moscow, 127276, Russia K.A. Timiryazev Institute of Plant Physiology, Russian Academy of Sciences Moscow Russia

**Keywords:** DNA barcode, molecular phylogeny, taxonomy, Ranunculales, ITS2, *rbc*L, *mat*K

## Abstract

We studied the allopatric complex *Pulsatilla patens* (L.) Mill. s.lat. (Ranunculaceae) in north-eastern European Russia and the Urals. In this region, there are two kinds of *P. patens* with different perianth colours in monochrome and polychrome populations. To clarify their taxonomic boundaries, we used the sequences of chloroplast DNA (*rbc*L and *mat*K) and nuclear DNA (ITS2), in addition to morphological characteristics. The combination of three markers (*rbc*L+*mat*K+ITS2) was found to be the most effective for phylogenetic resolution. The samples of two morphologically-different taxa *P. uralensis* and *P. patents* s.str. were shown to form a single clade on the phylogenetic tree. Based on the molecular phylogenetic analysis, we were not able to unequivocally prove the independent existence of *P. uralensis*.

## Introduction

The genus *Pulsatilla* Mill., which is sometimes included in *Anemone* s.lat. (e.g. Hoot et al. 1994, 2012), comprises more than 30 taxa which, as a rule, form intricate species complexes with a high degree of morphological variability (Stepanov 2014; Li et al. 2019; Sramko et al. 2019). The general classification, boundaries and the number of species and lower taxa have been revised several times, but no consensus has yet been reached (e.g. Tamura 1995; Tzvelev 2012; Grey-Wilson 2014). Furthermore, taxa of *Pulsatilla* often hybridise with each other in common habitats (Akeroyd 1993; Bakin 2005; Stepanov 2014), which makes it difficult to define the species’ boundaries. Many species and subspecies of *Pulsatilla* are rare and subject to protection (Holub and Prochazka 2000; Council of the European Union 2007; Bardunov and Novikov 2008; The IUCN Red List of Threatened Species 2019). In the Russian Federation, even widespread *Pulsatilla* species are subject to protection due to the small number of habitats, small population sizes and high anthropogenic impact. Fourteen species of *Pulsatilla* are recorded in the European part of Russia (Tzvelev 2001) and 11–15 in the Asian part (Malyshev 2012; Timokhina 1993). In addition, there are several questions regarding the identification and size of taxa within the *P. patens* s.lat. complex, despite several recent studies on molecular phylogenetic relationships in the genus (Li et al. 2019; Sramko et al. 2019).

In the Urals and the adjacent parts of the Russian Plain, four taxa of Pulsatilla ser. Patentes can be found: *P. patens* s.str., *P. uralensis* (Zamelis) Tzvelev, *P. multifida* (Pritz.) Juz. and *P. angustifolia* Turcz. (Tzvelev 2001, 2012). The main diagnostic characteristics used to recognise taxa within *P. patens* s.lat. are the colour of the perianth, the degree of dissection of the leaf blade (i.e. number of teeth), the presence and length of the petiolule of the apical segment and the width of the apical segment (Juzepczuk 1937; Tzvelev 2001; Egorova et al. 2017). All of them have extensive ranges of distribution and different centres of speciation, but only *P. patens* s.str. and *P. uralensis* are found in the Komi Republic. The European *P. patens* s.str. (= P. patens subsp. patens) only slightly expands beyond the boundaries of Europe. It is mainly distributed on the western macro-slope of the Urals (Central and Southern Urals) and also extends into Siberia and Central Asia (Tzvelev 2001; Sushentsov 2008). In Europe, this taxon is morphologically fairly uniform, but becomes extremely polymorphic in the Urals and Siberia (Juzepczuk 1937). *Pulsatilla uralensis* is distributed in the Central and Southern Urals (Kulikov 2005; Sushentsov 2008) and the adjacent part of the Russian Plain, in the basin of the Vyatka River (Egorova et al. 2017).

Due to the past separate geographical range, which suggests allopatric geographic isolation, and nomenclature confusion the circumscription of some species is a matter of much debate. For instance, *P. uralensis* (Zamelis) Tzvelev (= P. patens subsp. uralensis Zamelis) is often used synonymously with *P. flavescens* (Zucc.) Juz. [nom. illeg., non-Boros = P. patens subsp. flavescens (Zucc.) Zamelis]. However, we consider these taxa to be conspecific and, despite the widespread use of both names in regional floristic surveys and databases (see The Plant List 2020; World Flora Online 2020; NCBI 2020), the name *P. flavescens* is illegitimate (Somlay 2000). Therefore, in this study, we will use the commonly-accepted name *P. uralensis* (Zamelis) Tzvelev (see Tzvelev 2001, 2012; The Euro+Med PlantBase 2020) in order to also avoid confusion with the homonym of the species.

To date, no particular research of *Pulsatilla* species in the allopatric zone in the northern part of European Russia (within 59°12'–68°25'N and 45°25'–66°15'E) has been carried out. The northern boundary of the *Pulsatilla* range passes through the Komi Republic, while over 100 localities of plants of different coloured flowers are included under *P. patens* in the Red Book for the region (Martynenko 2009). The location of the region in the allopatric zone of European *P. patens* s.str. and Ural-Siberian *P. uralensis*, plus the elevated degree of polymorphism of diagnostic characteristics (i.e. colour of the perianth and dissection of the leaf blade) indicate the need to supplement the morphological methods with modern molecular genetic studies. Therefore, the aims of this work were to: 1) identify the taxa of *P. patens* s.lat. in north-eastern European Russia using herbarium specimens and wild populations; and 2) describe their phylogenetic relationships using plastid markers (*mat*K and *rbc*L) and a nuclear marker (ITS2) recommended for plants by the DNA Barcode consortium.

## Materials and methods

**Figure 1.**
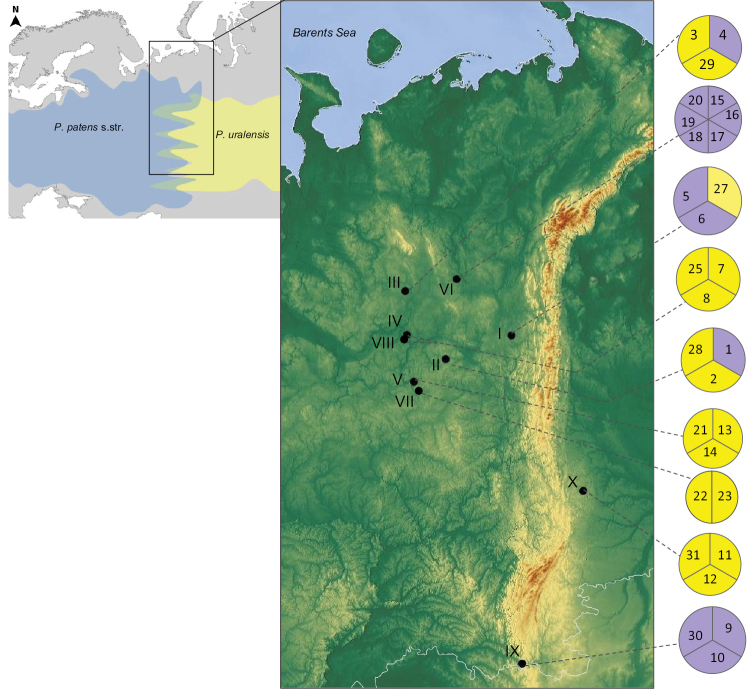
Distribution map of the sampling sites of *P. patens* s.str. and *P. uralensis* in north-eastern European Russia and the Urals. The colour on the diagrams indicates the colour of the perianth in different taxa: blue-violet – *P. patens* s.str., yellow – *P. uralensis*. The locations (I–X) and samples correspond to Table 2.

The study area is located in north-eastern European Russia (Fig. 1). Thirty-one samples from ten populations were collected by the authors from the Komi Republic, Orenburg Oblast and Sverdlovsk Oblast as detailed in Table 1. Most samples were collected from the Komi Republic within 59°12'–68°25'N and 45°25'–66°15'E. In each population, generative specimens were collected during the flowering period, at least 5–15 m apart from each other. We collected: 1) nine samples from hybridogenous polychrome populations (i.e. I, II and III) with yellow or blue-violet flowers; 2) eleven samples of *P. uralensis* from monochrome populations (i.e. IV, V, VII, VIII and X) with yellow (from pale yellow to deep yellow) and occasionally with white flowers; and 3) nine samples of *P. patens* s.str. from monochrome populations (i.e. VI and IX) with blue-violet flowers (Table 1).

**Table 1.** The studied *Pulsatilla* populations, characteristics of the samples, and GenBank and BOLD Systems numbers.

Population number	Geographic location/Habitat/ Coordinates	Population characteristics*	Taxon	Perianth colour	Sample number	GenBank accession number			BOLD Systems sample ID
						*rbc*L	*mat*K	ITS2	
I	Russia, Komi Republic, Troitsko-Pechorsky District, near settlement of Znamenka, 2 km to the east, right bank of the Pozheg River / Shrub - green moss - lichen pine forest / 61.9670°N, 56.8894°E	Hybridogenous polychrome population of P. patens s.str. × uralensis	*P. patens* s.str.	blue-violet	5	MK050017	MK050048	MK424550	SYKO-PV-17508
			*P. uralensis*	yellow	6	MK050018	MK050049	MK424551	SYKO-PV-17512
			*P. patens* s.str	blue-violet	27	MK050019	MK050050	MK424552	SYKO-PV-17524
II	Russia, Komi Republic, Ust-Kulomsky District, near settlement of Nizhny Yarashyu, valley of the Vychegda River / Lichen pine forest / 62.0927°N, 54.2982°E	Hybridogenous polychrome population of P. patens s.str. × uralensis	*P. patens* s.str.	blue-violet	1	MK050020	MK050051	MK424553	SYKO-PV-17509
			*P. uralensis*	yellow	2	MK050021	MK050052	MK424554	SYKO-PV-17526
			*P. uralensis*	yellow	28	MK050022	MK050053	MK424555	SYKO-PV-17525
III	Russia, Komi Republic, Knyazhpogostsky District, near the settlement of Meshchura, valleys of the Vym and Elva Rivers / Green moss - lichen pine forest / 63.3375°N, 50.9150°E	Hybridogenous polychrome population of P. patens s.str. × uralensis	*P. uralensis*	yellow	3	MK050023	MK050054	MK424556	SYKO-PV-17510
			*P. patens* s.str.	blue-violet	4	MK050024	MK050055	MK424557	SYKO-PV-17511
			*P. uralensis*	yellow	29	MK050025	MK050056	MK424558	SYKO-PV-17532
IV	Russia, Komi Republic, Syktyvdinsky District, near settlement of Kocchoyag, 300–400 m north of station Yazel / Railway mound / 61.9588°N, 50.6117°E	Monochrome population of *P. uralensis*	*P. uralensis*	yellow	7	MK050026	MK050057	MK424559	SYKO-PV-17513
			*P. uralensis*	yellow	8	MK050027	MK050058	MK424560	SYKO-PV-17527
V	Russia, Komi Republic, Koigorodskiy District, near settlement of Vezhye (Uzhga-2) / Lichen pine forest / 60.6284°N, 51.0303°E	Monochrome population of *P. uralensis*	*P. uralensis*	yellow	13	MK050034	MK050065	MK424567	SYKO-PV-17535
			*P. uralensis*	yellow	14	MK050035	MK050066	MK424568	SYKO-PV-17536
			*P. uralensis*	yellow	21	MK050036	MK050067	MK424569	SYKO-PV-17531
VI	Russia, Komi Republic, Ukhtinsky District, near settlement of Shudayag, theTiman limestones / Cowberry- green-moss pine forest / 63.5199°N, 53.5949°E	Monochrome population of *P. patens* s.str.	*P. patens* s.str.	blue-violet	15	MK050037	MK050068	MK424570	SYKO-PV-17516
			*P. patens* s.str.	blue-violet	16	MK050038	MK050069	MK424571	SYKO-PV-17517
			*P. patens* s.str.	blue-violet	17	MK050039	MK050070	MK424572	SYKO-PV-17518
			*P. patens* s.str.	blue-violet	18	MK050040	MK050071	MK424573	SYKO-PV-17519
			*P. patens* s.str.	blue-violet	19	MK050041	MK050072	MK424574	SYKO-PV-17520
			*P. patens* s.str.	blue-violet	20	MK050042	MK050073	MK424575	SYKO-PV-17521
VII	Russia, Komi Republic, Koigorodskiy District, near settlement of Vezhye (Uzhga-1) / Lichen pine forest / 60.6005°N, 50.9959°E	Monochrome population of *P. uralensis*	*P. uralensis*	yellow	22	MK050043	MK050074	MK424576	SYKO-PV-17522
			*P. uralensis*	yellow	23	MK050044	MK050075	MK424577	SYKO-PV-17530
			*P. uralensis*	yellow	24	–	–	–	–
VIII	Russia, Komi Republic, Syktyvdinsky District, near settlement of Kocchoyag / Lichen pine forest / 61.9428°N, 50.6281°E	Monochrome population of *P. uralensis*	*P. uralensis*	yellow	25	MK050046	MK050077	MK424579	SYKO-PV-17523
			*P. uralensis*	yellow	26	–	–	–	–
IX	Russia, Orenburg Oblast, Gaysky District, near village of Khmelevka, slope facing to east / Steppe meadow / 51.13°N, 57.54°E	Monochrome population of *P. patenss.str.*	*P. patens* s.str.	blue-violet	9	MK050028	MK050059	MK424561	SYKO-PV-17514
			*P. patens* s.str.	blue-violet	10	MK050029	MK050060	MK424562	SYKO-PV-17528
			*P. patens* s.str.	blue-violet	30	MK050030	MK050061	MK424563	SYKO-PV-17533
X	Russia, Sverdlovsk Oblast, near city of Rezh, carbonate rocks / Pine forest / 57.23°N, 61.25°E	Monochrome population of *P. uralensis*	*P. uralensis*	yellow	11	MK050031	MK050062	–	SYKO-PV-17515
			*P. uralensis*	yellow	12	MK050032	MK050063	MK424565	SYKO-PV-17529
			*P. uralensis*	yellow	31	MK050033	MK050064	MK424564	SYKO-PV-17534

Note: * polychrome populations are formed by plants with yellow and blue-violet flower colours of varying intensity; monochrome populations are formed only by plants with a yellow flower colour or only a blue-violet flower colour of varying intensity.

**Figure 2.**
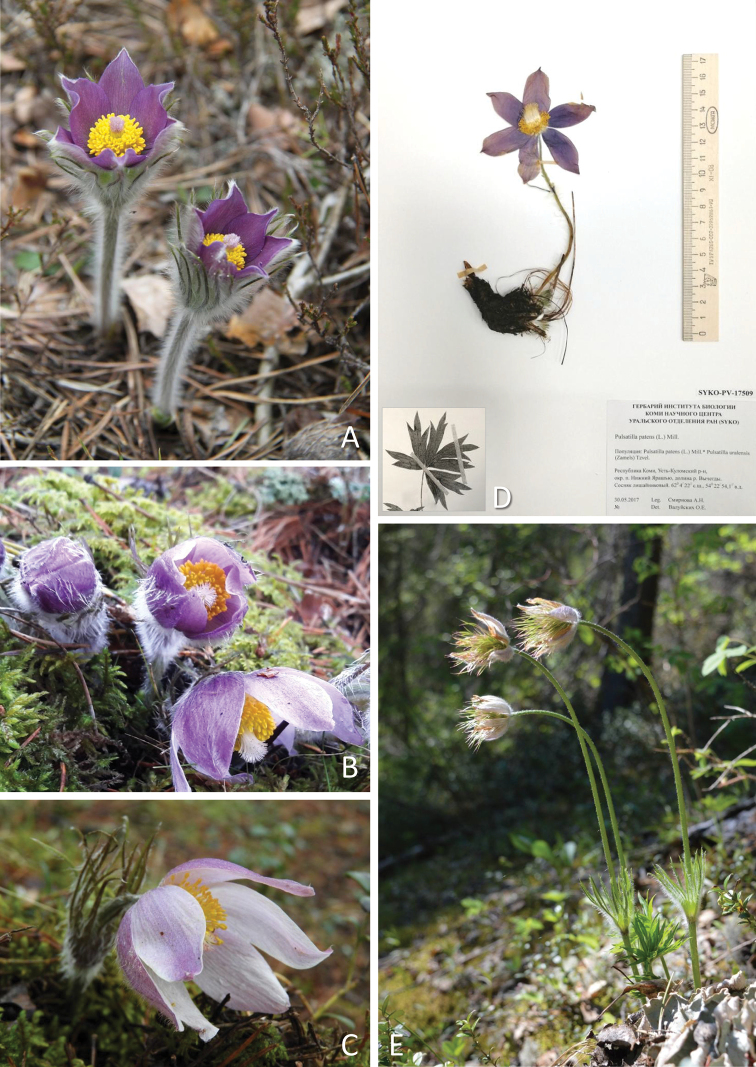
*Pulsatilla patens* s.str. (L.) Mill. (P. patens subsp. patens) **A–C** flowers with different perianth colour **D** herbarium specimen of a flowering shoot and typical leaf blade **E** plant just after flowering with unripe fruits. The photographs show sample number 5 (**A, D**), sample number 15 (**B**) and sample number 18 (**C**).

**Figure 3.**
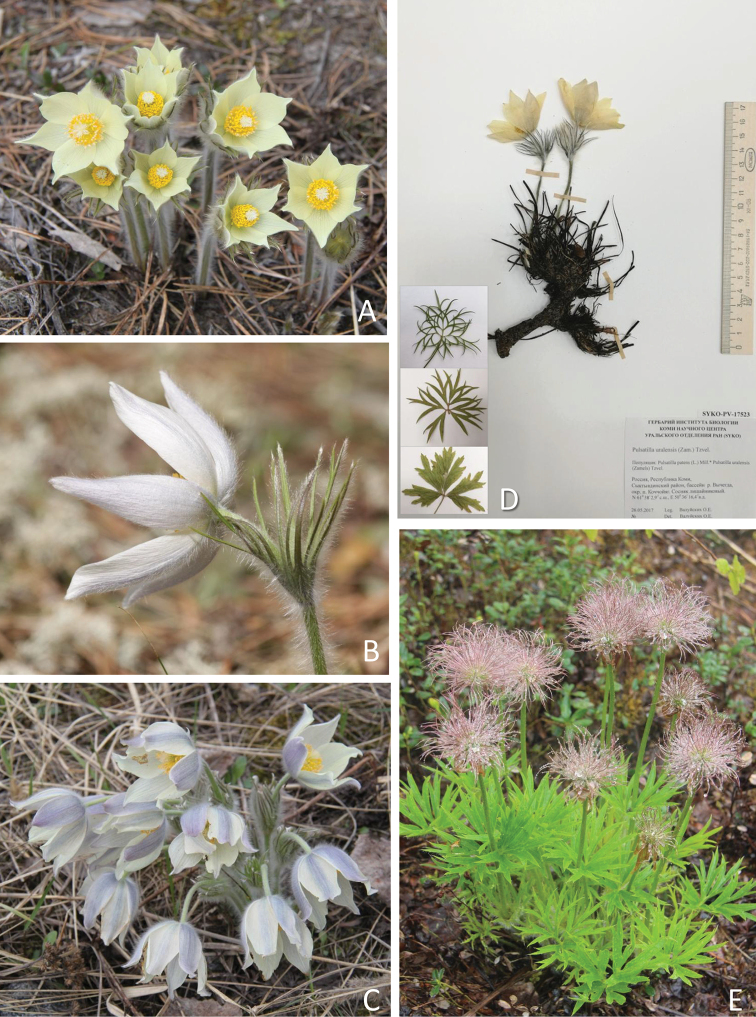
*Pulsatilla uralensis* (Zamelis) Tzvelev **A–C** flowers with different perianth colour **D** herbarium specimen of a flowering plant and different leaf blades **E** fruiting plant. The photographs show sample number 6 (**B**), sample number 7 (**C**) and sample number 25 (**D**).

Furthermore, we analysed over 120 specimens of *P. patens* s.lat. from the Herbarium of the Institute of Biology of the Komi Scientific Center of the Ural Branch of the Russian Academy of Sciences (**SYKO**). According to morphological characteristics, these plants were identified as *P. patens* s.str. (Fig. 2) or *P. uralensis* (Fig. 3). *Pulsatilla patens* s.str. has blue-violet flowers, while *P. uralensis* has pale yellow to yellow flowers. The main morphological differences between *P. patens* s.str. and *P. uralensis* are summarised in Table 2.

**Table 2.** Morphological comparison between *P. patens* s.str. and *P. uralensis*.

**Characters**	***P. patens* s.str.**	***P. uralensis***
Flower colour	Blue-violet (different saturation)	Yellow (white or light yellow)
Lobes of basal leaves	≤ 26 (17–30)	≥ 26 (22–43)
Width of leaf-lobes	0.3–0.6 cm	0.2–0.5 cm
Stalk of the central lobe of the basal leaves	Absent	0.7–1.2 cm / absent
Flowering time	Early May to early June	Early May to early June

### DNA extraction, amplification and sequencing

Sequences of plastid DNA (*rbc*L and *mat*K) and nuclear DNA (ITS2) have been successfully used for plant identification and DNA barcoding in various taxonomic groups (Cai et al. 2010, Wattoo et al. 2016, Saddhe Kumar 2017), including the genus *Pulsatilla* (Li et al. 2019). ITS2 is considered to be the most effective barcode for the identification of more than half of *Pulsatilla* species (Li et al. 2019). Therefore, we tested the ability of DNA barcoding to distinguish between the taxa of *P. patens* s.lat. Thirty-one samples were analysed and three barcode regions (*rbc*L, *mat*K and ITS2) were amplified, sequenced and aligned.

Total genomic DNA was isolated from dried leaves using the Sorb–GMO–B kit (Sintol, Russia) in accordance with the manufacturer’s instructions. PCR fragments were amplified in 50 μl of a mixture containing 10 μl of Screen Mix (Eurogen, Russia), 10 μl of each primer (0.3 μM) (Eurogen, Russia), 18 μl of ddH_2_O (Ambion, USA) and 2 μl of DNA template (1÷100 ng). The ITS2 sequences were amplified with universal primers ITS-5 (5'–GGAAGTAAAAGTCGTAACAAGG –3') and ITS-4 (5'–TCCTCCGCTTATTGATATGC– 3'); the *rbc*L and *mat*K sequences – with primers SI_For (5'– ATGTCACCACAAACAGAGACTAAAGC –3'), SI_Rev (5'–GTAAAATCAAGTCCACCRCG–3') and KIM 3F (5'–CGTACAGTACTTTTGTGTTTACGAG –3'), KIM 3R (5'–ACCCAGTCCATCTGGAAATCTTGGTTC–3'), respectively (Kress et al. 2009). Thermal cycling included heating to 95 °C for 4 min, followed by 34 cycles of 60-s melting at 95 °C, 30-s annealing at 50 °C (for *rbc*L), 55 °C (for ITS2), 61 °C (for *mat*K) and 40-s extension at 72 °C, with a final extension for 5 min at 72 °C. PCR and sequencing were carried out using the equipment of the Center for Collective Usage «Molecular Biology» of the Institute of Biology of the Komi Scientific Center of the Ural Branch of the Russian Academy of Sciences (Syktyvkar, Russia).

### Phylogenetic analysis

Multiple alignments of nucleotide sequences were obtained using ClustalW in the MegaX programme (Thompson et al. 1994, Kumar et al. 2018). There were some missing data (see Table 2) and the alignments of the *rbc*L, *mat*K loci and the ITS2 region were analysed separately and in concatenation (*rbc*L+*mat*K+ITS2).

Phylogenies were constructed based on the GTR+Г+I model for all alignments using the Bayesian Inference (BI) and Maximum Likelihood (ML) analysis. The BI analysis was conducted using MrBayes-3.2.5 (Ronquist and Huelsenbeck 2003). Three “hot” and one “cold” Markov chains were run for 1 × 10^6^ cycles in two repetitions with the selection of each 200^th^ generated tree. The phylogenetic tree and the probabilities of its branching were obtained after discarding the first 25% of the model for estimating the parameters of nucleotide substitutions and their probabilities. The ML analysis was performed using the MegaX programme (Kumar et al. 2018) with bootstrap analysis from 1,000 replicas. Graphical viewing and editing of trees were carried out in the programmes Fig. Tree (ver. 1.4.2) and Adobe Photoshop CC (19.0).

In the analysis, we used nucleotide sequences obtained by us or taken from the NCBI database (GenBank) and BOLD Systems (accessions numbers on phylogenetic trees are indicated in Table 2). Representatives of some species of *Anemone*, *Anemoclema*, *Clematis* and *Hepatica* were used as an external group. In this study, we discuss the phylogenetic hypotheses obtained from each individual dataset separately (*rbc*L, *mat*K and ITS2) and analysis of the combined dataset of all three markers. All new *rbc*L, *mat*K and ITS2 sequences, obtained by us and used in this study, were deposited to the GenBank (accession No. MK050017–MK050077, MK424550–MK424579) and Barcode of Life databases (BOLD Systems sample ID: SYKO-PV-17508–SYKO-PV-17536) (Table 2). Other *Pulsatilla* species from the BOLD Systems (BOLD Systems 2020) and GenBank (NCBI 2020) databases that had all three sequences (*rbc*L, *mat*K and ITS2) were used in phylogenetic analysis.

## Results

The data matrix of *rbc*L sequences included 486 bp, *mat*K – 775 bp and ITS2 – 214-215 bp. The concatenated data matrix of *rbc*L+*mat*K+ITS2 sequences included 1,476 bp.

**Figure 4.**
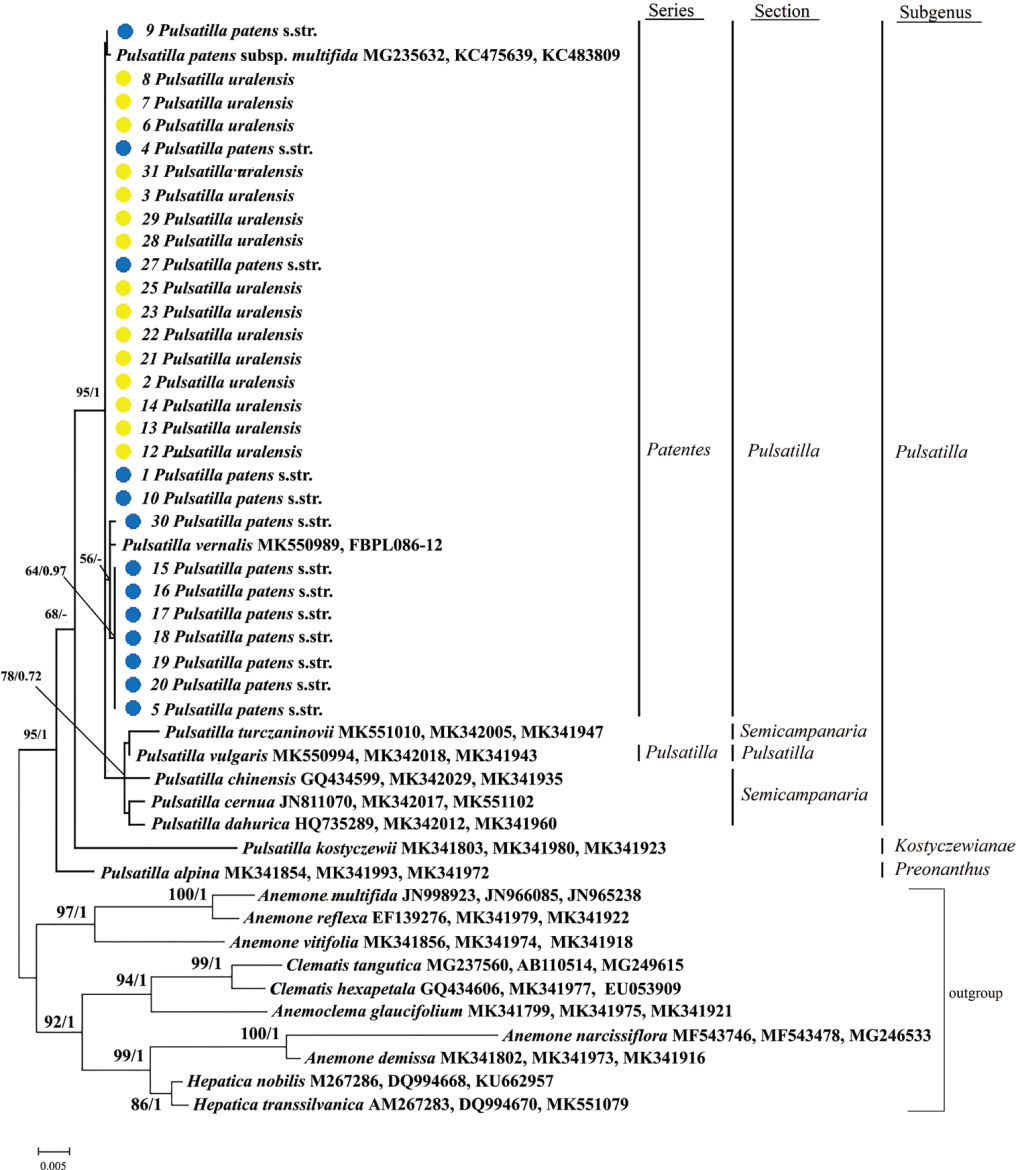
Combined Maximum Likelihood (ML) and Bayesian Inference (BI) phylogenetic tree (*rbc*L+ *mat*K+ITS2) of 37 *Pulsatilla* samples and 10 outgroup samples. All new 28 samples of *P. patens* s.str. and *P. uralensis* are marked with dots. Outgroups include *Anemone*, *Anemoclema*, *Clematis* and *Hepatica* species. ML bootstrap support (left) and BI posterior probability (right) are recorded along branches. Values below 50% are not shown.

We reconstructed molecular phylogenetic trees using BI and ML analyses and obtained similar topologies for concatenated dataset trees (*rbc*L, *mat*K and ITS2), including 37 terminals of *Pulsatilla* species and ten outgroups (Fig. 4). *Pulsatilla alpina* (subgenus Preonanthus) and *P. kostyczewii* (subgenus Kostyczewianae) were the first two splits within the genus *Pulsatilla*, respectively. All closely-related taxa from P. section Pulsatilla (*P. patens* s.str., *P. uralensis*, P. patens subsp. multifida, *P. vernalis* and *P. vulgaris*) and P. section Semicampanaria (*P. turczaninovii*, *P. chinensis*, *P. cernua* and *P. dahurica*) of the subgenus Pulsatilla belong to a clade with a high support (95% BS and 1 PP). *P. vulgaris* and the section Semicampanaria belong to a clade with an elevated bootstrap value (78% BS and 0.72 PP). *Pulsatilla patens* s.str. (samples No. 5, 15–20 and 30) and *P. vernalis* belong to a clade with a low bootstrap value (56% BS). We were not able to establish relationships for the remaining samples of *P. patenss.str.* (sample numbers 1, 4, 9, 10 and 27) and *P. uralensis* (sample No. 2, 3, 6, 7, 8, 12–14, 21–23, 25, 28, 29 and 31) from different geographic locations (Table 2), since they arose as terminals from a polytomy.

Trees that were constructed based on the sequences of single genes had a low resolution. On the phylogenetic tree of the plastid region *rbc*L, all species of the subgenus Pulsatilla were also united into one clade (see Suppl. material 1). The exceptions are the apical clade represented by two sequences of *P. cernua* and *P. chinensis* (63% BS and 0.91 PP) and the subclade represented by eight sequences of *P. patens* s.str. (samples No. 5, 15–20 and 30) and *P. vernalis* (FBPL086-12) (65% BS and 0.82 PP). The molecular analysis of the sequences showed that samples of *P. patens* s.str. (No. 5, 15–20 and 30) differ from the others by one variable site in the *rbcL* region (A/G_61_) (see Suppl. material 2). Comparison of the *mat*K and ITS2 sequences could not separate *P.* subgenera *Pulsatilla*, *Kostyczewianae* and *Preonanthus* (see Suppl. material 3, 4). On the *matK* phylogenetic tree, the same clade is distinguished, including eight blue-flowered samples of *P. patens* s.str. (5 and 15–20), however, with low support (0.67 PP). For such samples, one variable site (C/T_472_) was identified (Suppl. material 2).

## Discussion

Global climate perturbations throughout the Quaternary period caused active migrations of *Pulsatilla* within Eurasia, followed by secondary polyploidy and increased polymorphism (Sramko et al. 2019). The ancestor of the modern *P. patens* s.lat. populations inhabiting north-eastern European Russia most likely grew in the steppes of Eastern Eurasia (Sramko et al. 2019). During the Pleistocene, the ancestor of the modern *P. patens* s.lat., perhaps, together with the southern Siberian forest-steppe species, spread to the west and further north along the Ural Mountains (Knyazev et al. 2007, Kulikov et al. 2013). Climate change associated with the Quaternary period contributed significantly to the diversification of *P. patens* s.lat. The disruption of the continuous distribution of species by the Pleistocene glaciations led to geographical disjunction and formation of a number of allopatric morphological forms in the European and Asian parts of the range of the species (Bobrov 1944; Tzvelev 2001; Ronikier et al. 2008; Kricsfalusy 2015 etc.). During the post-glacial colonisation, the ranges of migrants from several refugia started to touch or overlap in contact zones.

A part of the territory of north-eastern European Russia, including the Komi Republic, was covered by the last Late Pleistocene glaciations (Andreicheva 2002; Ilchukov 2010) and many of the known *Pulsatilla* locations in this region are the result of post-glacial colonisation. On the northern limit of the range, the most typical habitats for *P. patens* s.lat. are arid pine and mixed forests, forest clearings and edges. Large populations (up to 500–1,000 and more specimens) in the region are rare and are confined to lichen forests in the southern part of the Komi Republic (60–62°N, 51–54°E). During the Pleistocene, climate changes led to repeated Meridional displacements of vegetation zones (Andreicheva 2002). Evidence of these processes is provided by the fragments of a relict petrophytic floristic complex that currently exists on limestones in the north-east of European Russia, including the Timan limestones (Yudin 1963; Teteryuk et al. 2006). Small and isolated populations of *P. patens* s.lat. (up to 150–200 specimens) survived at the outcroppings of bedrocks; they grow together with the species of the relict petrophytic floristic complex in the river valleys of the Timan limestones (63–64°N, 52–53°E). Therefore, it is likely that some populations of *P. patens* s.str. (e.g. population VI), together with some species of bushes and herbaceous plants, survived during the last glaciations and are the part of more ancient vegetation compared to the adjacent flatland ecosystems that formed after the last ice-sheet glaciations.

Molecular phylogenetic analyses showed that all samples of *P. patens* s.str. and *P. uralensis* belong to a single clade and some groups within it arose with moderate statistical support, which makes it impossible to draw more definitive conclusions. Such low resolution is generally associated with *Pulsatilla* species (Li et al. 2019) and with specific factors related to the evolution of the species (by hybridisation and polyploidisation). Therefore, our discussion will be carefully constructed, given the low resolution that we obtained.

The obtained results of the molecular phylogenetic analysis (nuclear and chloroplast loci) are not consistent with the traditional morphological approach, according to which the isolation of *P. patens* s.str. and *P. uralensis* is based on the colour of the perianth – the distinctive feature on the level of species (Juzepczuk 1937; Tamura 1995; Tzvelev 2012; Egorova et al. 2017; amongst others). Only a small part of the samples belongs to a subclade (Fig. 4) that is represented exclusively by blue-flowered samples of *P. patens* s.str. (64% BS and 0.97 PP). This small subclade includes only blue-flowered *P. patens* s.str. (5, 15–20) from the Komi Republic, *P. patens* s.str. (30) from the Orenburg Oblast of monochrome populations and European plant *P. vernalis* (MK550989, FBPL086-12). Such a relationship of the complex of *P. patenss.lat.* and *P. vernalis* with erect flowers and less dissected leaf blades was found by Sramko et al. (2019), who suggested to include *P. vernalis* into Series *Patentes*. Our phylogenetic trees showed that *P. vernalis* is close to *P. patens* s.str. (Fig. 4). These species are very different in morphology (e.g. *P. vernalis* has basal overwintering leathery leaves) and the range of *P. vernalis* does not currently cover north-eastern European Russia. It is an alpine species which, during the Ice Age in Europe, was widespread in the lowlands in sparse pine forests and heathlands. It is currently preserved in the Scandinavian refugia (Ronikier et al. 2008). Hybridisation and/or introgression between the ancestor of the modern forms of *P. patens* s.str. and *P. vernalis* may have occurred a long time ago during speciation.

According to our phylogenetic results, all the populations from different locations studied (except for population VI) belong to the same species. Even though ITS2, *mat*K and *rbc*L were found to be suitable regions for barcoding in species of *Pulsatilla*, in our study, they did not allow us to isolate *P. uralensis* from monochrome populations in north-eastern European Russia and the Urals into a separate group. The absence of divergence in the tree (see Fig. 4) may indicate the hybrid origin of all yellow-flowered samples and the hybrid origin of modern *P. uralensis* populations. Our results indicate recent speciation and incomplete lineage sorting, resulting in very few accumulated genetic differences. This study suggests that *P. uralensis* should not be recognised as a separate species, despite its Ural-Siberian detached range, yellow flowers, and finer leaf lobes. Despite being morphologically identified by the colour of its perianth and leaf structure, our phylogenetic results do not support this distinction. Using the ITS2, matK and rbcL markers, we found only limited phylogenetic resolution within the P. subgenus Pulsatilla. Molecular data showed that the yellow-flowered taxon should be considered a subspecies within the *P. patens* s.lat. complex. At least in north-eastern European Russia and in the Urals, where its west distribution border passes, this taxon does not separate clearly from *P. patens* s.str. It is possible that the standard DNA barcode markers, ITS2, *mat*K and *rbc*L, are not applicable within the *P. patens* complex and do not allow us to assess the divergence between these closely-related taxa.

Given the high morphological variability of *P. patens* s.lat. and the widespread presence of hybridogenic populations in the Urals in the Orenburg and Sverdlovsk Oblasts (Kulikov 2005, Sushentsov 2008) and the vicinity of the Komi Republic on the Russian Plain (Egorova et al. 2017), we can expect that many plants in the zone of overlapping of their ranges are hybrids. The morphological analysis also supports the opinion that *P. uralensis* is polymorphic with high variability of leaf characteristics (Sushentsov 2008). In order to clarify the species’ limits of *P. uralensis*, a more detailed study with different methodological approaches is required, involving data on closely-related taxa from a wider area from Siberia and other parts of their range. This is beyond the scope of this study.

An indication of hybrid processes in *Pulsatilla* populations is their polychromy (Bobrov 1944). In greenhouse experiments, the hybrid progeny of *P. patens* s.lat. most often has yellow, sometimes white and blue and rarely pink perianth (at a ratio of 4:2:1) (Pavlova 1990). Taking into account the prevalence of polychrome and monochrome yellow-flowered populations of *Pulsatilla* in the Komi Republic, we believe that an intricate complex with a significant participation of hybrid forms has formed in this territory, whereas the populations of *P. patens* s.str. have limited distribution (e.g. population VI). It is possible that there is an active process of introgression and “supplanting” of the European blue-flowered *P. patens* s.str., followed by the “absorption” of this species by the Ural-Siberian yellow-flowered *P. uralensis*.

The obtained data are consistent with the results of the study of the population variability of morphological features of *Pulsatilla* in the adjacent territory (Sushentsov 2008; Egorova et al. 2017). To the south, in the basin of the Vyatka River (57–58°N), we identified populations of *P. patens* s.str., as well as hybridogenous populations of P. patens s.str. × uralensis (Egorova et al. 2017). They occupy a different ecological niche, which is preserved within the territory of the Komi Republic. Yellow–flowered populations are common on the pine terraces in lichen pine forests. Such conditions (sandy biotopes favourable for cross-pollination and seed renewal with reduced competition from other plants) contribute to the manifestation of *Pulsatilla* polymorphism in hybridizsation zones. In the large monochrome yellow-flowered population No. V of *P. uralensis* in the pine forests of lichen type in the south of the republic, we found specimens with highly dissected leaves (samples No. 13, 14 and 21) that were also included in the clade of phylogenetic trees, together with the hybrid forms and *P. patens* s.str. Leaf-blade lobes of *P. uralensis* are finely dissected and the ultimate lobes (up to 43 pieces) are linear-lanceolate to narrowly linear, the central leaflet has an 8–12 mm petiolule.

Thus, using the sequences of chloroplast (*mat*K, *rbc*L) and nuclear DNA (ITS2), we showed that, in north-eastern European Russia and the Urals, populations with yellow and blue-violet flowers belong to the same species (*P. patens* s.lat.). Isolated monochrome populations of *P. patens* s.str. with blue-violet flowers are preserved within the territory of the Komi Republic and have a limited distribution range.

Since there are still many questions about the taxonomy of *P. patens* s.lat. complex, we believe that, in order to improve the ability to distinguish closely-related taxa in this group, it is necessary1) to analsze a large number of plants with an accurate identification of the main diagnostic characteristics; to scrutinise additional morphological and micro-morphological characteristics; 2) to obtain information on the location of the studied samples in the species range (the centre or the border of the range); to obtain information on their possible hybridisation and introgression with other species of the genus *Pulsatilla* on this territory, taking into account the factor of overlapping and the relict nature of the communities; 3) to obtain information on the abundance, distribution range and genetic structure of populations. The use of DNA barcoding process, geographically-expanded sampling and analysis of the genetic structure of populations of closely related *Pulsatilla* species are necessary to reconstruct the phylogenetic relationships between taxa of series *Patentes*.

## Supplementary materials

Supplementary material 1 Maximum Likelihood and Bayesian Inference phylogenetic tree (rbcL) Data type phylogenetic tree Explanation note Maximum Likelihood and Bayesian Inference phylogenetic tree (*rbc*L) of 13 sequences of *P. patens* s.str. and 16 sequences of *P. uralensis*, plus the nine selected sequences in GenBank and BOLD Systems for different *Pulsatilla *species. Posterior probability and bootstraps are shown at the nodes, values below 50% are not shown. https://binary.pensoft.net/file/461973Olga E. Valuyskikh, Ludmila V. Teteryuk, Yana I. Pylina1, Oleg E. Sushentsov, Nikita A. Martynenko, Dmitry M. Shadrin

Supplementary material 2 Informative nucleotide sites in cpDNA (matK, rbcL) for *Pulsatilla* Data type molecular data https://binary.pensoft.net/file/461974Olga E. Valuyskikh, Ludmila V. Teteryuk, Yana I. Pylina1, Oleg E. Sushentsov, Nikita A. Martynenko, Dmitry M. Shadrin

Supplementary material 3 Likelihood and Bayesian Inference phylogenetic tree (matK) of 13 sequences of *P. patens* s.str. and 16 sequences of *P. uralensis* Data type phylogenetic tree Explanation note Maximum Likelihood and Bayesian Inference phylogenetic tree (*mat*K) of 13 sequences of *P. patens* s.str. and 16 sequences of *P. uralensis*, plus the nine selected sequences in GenBank and BOLD Systems for different species. Bootstraps and posterior probabilities are shown at the nodes, values below 50% are not shown. https://binary.pensoft.net/file/461975Olga E. Valuyskikh, Ludmila V. Teteryuk, Yana I. Pylina1, Oleg E. Sushentsov, Nikita A. Martynenko, Dmitry M. Shadrin

Supplementary material 4 Phylogenetic tree (ITS2) constructed using the Maximum Likelihood and Bayesian Inference of the 13 sequences of *P. patens* s.str. and 15 sequences of *P. uralensis* Data type phylogenetic tree Explanation note Phylogenetic tree (ITS2) constructed using the Maximum Likelihood and Bayesian Inference of the 13 sequences of *P. patens* s.str. and 15 sequences of *P. uralensis*, plus the nine selected sequences in GenBank and BOLD Systems for different species. Bootstraps and posterior probabilities are shown at the nodes, values below 50% are not shown. https://binary.pensoft.net/file/461976Olga E. Valuyskikh, Ludmila V. Teteryuk, Yana I. Pylina1, Oleg E. Sushentsov, Nikita A. Martynenko, Dmitry M. Shadrin
