# Supplementary figures and images for: Phylogenetic relationships and status of taxa of Pulsatilla uralensis and P. patens s.str. (Ranunculaceae) in north-eastern European Russia
Source: PhytoKeys. 2020 Oct 9;162:113–30. doi: 10.3897/phytokeys.162.53361 (PMC7578421; doi:10.3897/phytokeys.162.53361)

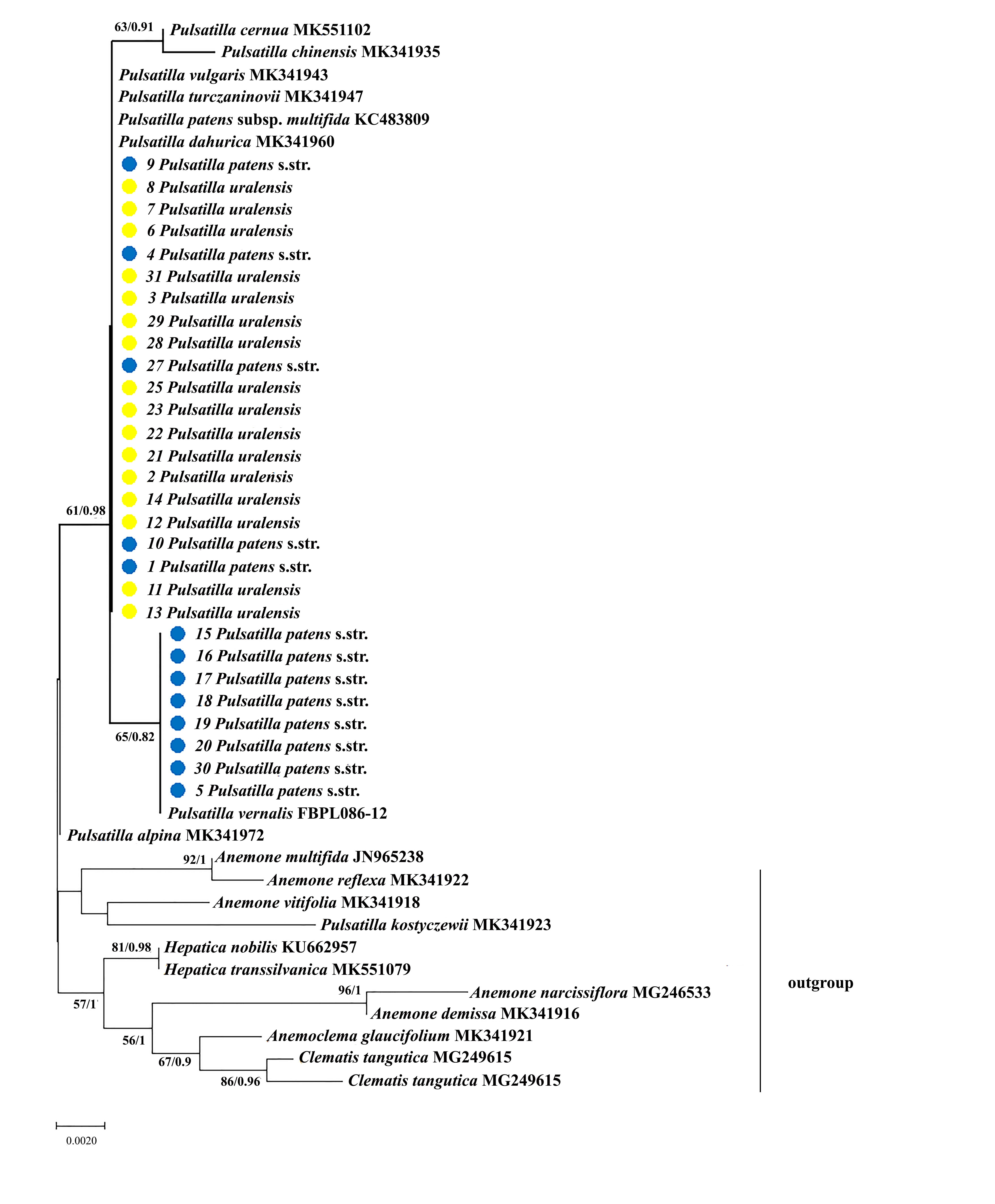

Supplement: Supplementary material 1 — Maximum Likelihood and Bayesian Inference phylogenetic tree (rbcL) [file phytokeys-162-113-s001.tif]

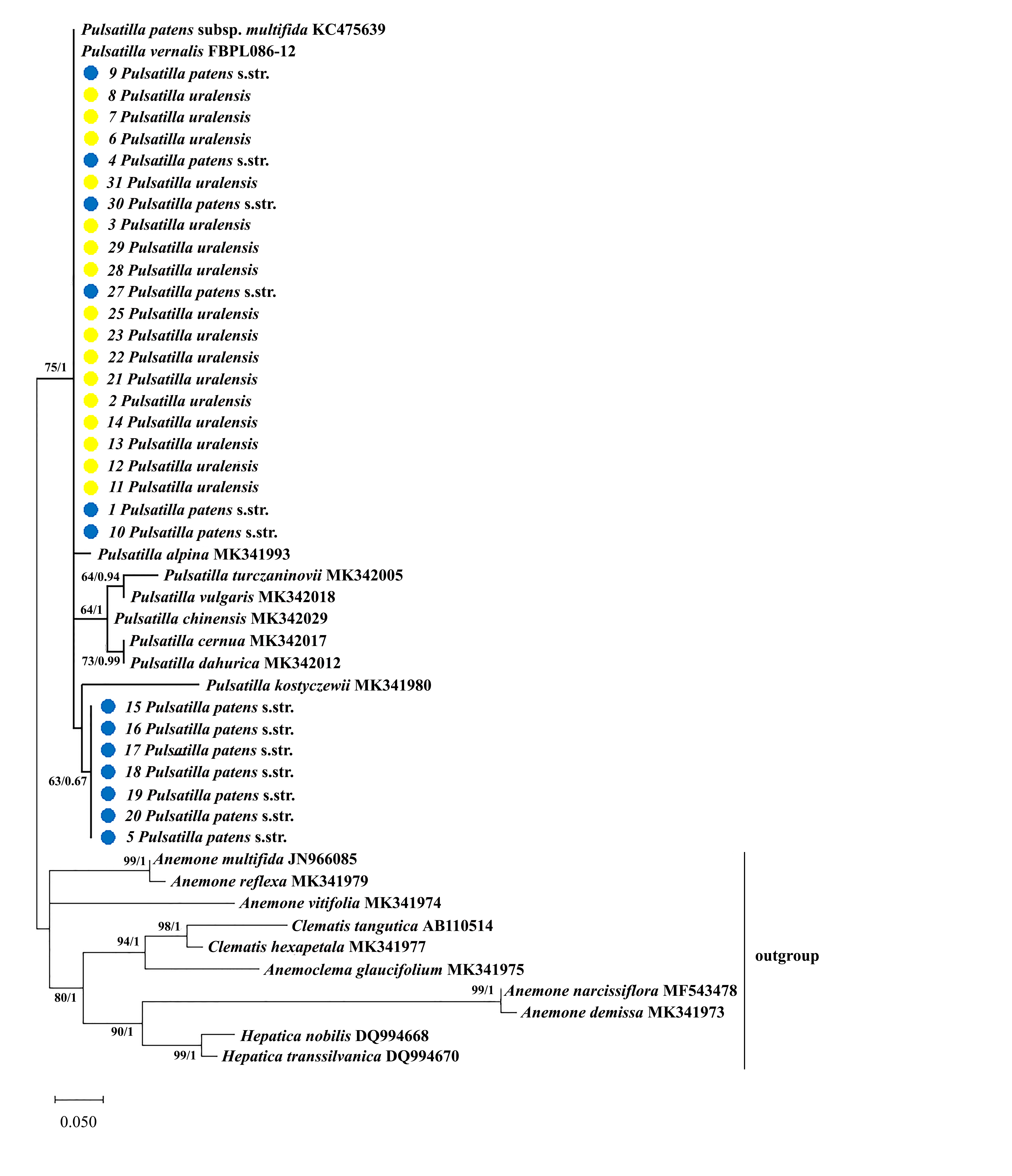

Supplement: Supplementary material 3 — Likelihood and Bayesian Inference phylogenetic tree (matK) of 13 sequences of P. patens s.str. and 16 sequences of P. uralensis [file phytokeys-162-113-s003.tif]

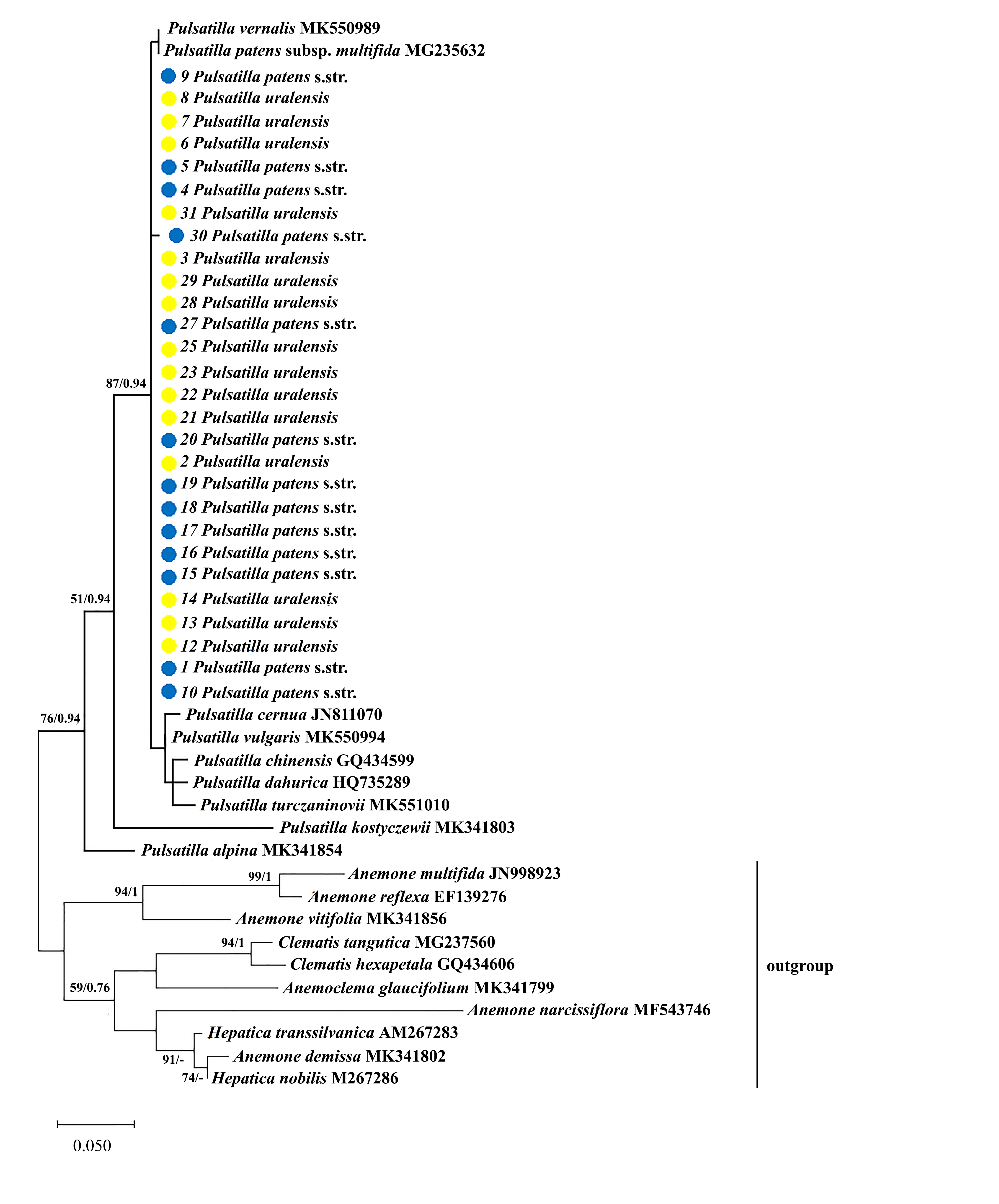

Supplement: Supplementary material 4 — Phylogenetic tree (ITS2) constructed using the Maximum Likelihood and Bayesian Inference of the 13 sequences of P. patens s.str. and 15 sequences of P. uralensis [file phytokeys-162-113-s004.tif]
