# Supplementary material for: Phylogenetic relationships and status of taxa of Pulsatilla uralensis and P. patens s.str. (Ranunculaceae) in north-eastern European Russia
Source: PhytoKeys. 2020 Oct 9;162:113–30. doi: 10.3897/phytokeys.162.53361 (PMC7578421; doi:10.3897/phytokeys.162.53361)
Supplement: Supplementary material 2 — Informative nucleotide sites in cpDNA (matK, rbcL) for Pulsatilla [file phytokeys-162-113-s002.doc]

Supplementary material 2. Informative nucleotide sites in cpDNA (*rbc*L, *mat*K) for *Pulsatilla*.

| Taxa*/ Nucleotide sites | *rbc*L position | *mat*K position |
| --- | --- | --- |
|  | 61 | 472 |
| Subgenus *Pulsatilla,* Section *Pulsatilla,* Series *Patentes* |  | |
| ***P. patens* s.str. (sample 1, 4, 9, 10 and 27)**  ***P. uralensis* (sample 2, 3, 6-8, 11-14, 21-23, 25, 28, 29 and 31)**  *P. patens* subsp. *multifida* KC483809, KC475639  ***P. patens* s.str. (sample 5, 15-20)**  ***P. patens* s.str. (sample 30)**  *P. vernalis* FBPL086-12 | **A** | **C** |
|  | **A** | **C** |
|  | A | C |
|  | **G** | **T** |
|  | **G** | **C** |
|  | G | С |
| Subgenus *Pulsatilla*, Section *Pulsatilla,* Series *Pulsatilla* |  | |
| *P. vulgaris* MK341943, MK342018 | A | A |
| Subgenus *Pulsatilla,* Section *Semicampanaria* |  | |
| *P. cernua* MK551102, MK342017 | A | C |
| *P. chinensis* MK341935, GQ434599 | A | C |
| Subgenus *Kostyczewianae* |  | |
| *P. kostyczewii* MK341923, MK341980 | A | A |
| Subgenus *Preonanthus* |  |  |
| *P. alpine* MK341972, MK341993 | A | C |

*According to Sramko et al. (2019). Our samples are highlighted in bold.
